# Correlations of gene expression with ratings of inattention and hyperactivity/impulsivity in tourette syndrome: a pilot study

**DOI:** 10.1186/1755-8794-5-49

**Published:** 2012-10-30

**Authors:** Yingfang Tian, Boryana Stamova, Bradley P Ander, Glen C Jickling, Joan R Gunther, Blythe A Corbett, Netty GP Bos-Veneman, Pieter J Hoekstra, Julie B Schweitzer, Frank R Sharp

**Affiliations:** 1 MIND Institute and Department of Neurology, University of California at Davis, 2805 50th St., Room 2434, Sacramento, CA, 95817, USA; 2 Laboratory of Gene Therapy, College of Life sciences, Shaanxi Normal University, Xi'an, Shaanxi, China; 3 MIND Institute and Department of Psychiatry, University of California at Davis, Sacramento, CA, USA; 4 Department of Psychiatry, University Medical Center Groningen, University of Groningen, Groningen, Netherlands

**Keywords:** Attention-deficit hyperactivity disorder (ADHD), Blood, RNA expression, Genomics, Microarray, Tourette syndrome

## Abstract

**Background:**

Inattentiveness, impulsivity and hyperactivity are the primary behaviors associated with attention-deficit hyperactivity disorder (ADHD). Previous studies showed that peripheral blood gene expression signatures can mirror central nervous system disease. Tourette syndrome (TS) is associated with inattention (IA) and hyperactivity/impulsivity (HI) symptoms over 50% of the time. This study determined if gene expression in blood correlated significantly with IA and/or HI rating scale scores in participants with TS.

**Methods:**

RNA was isolated from the blood of 21 participants with TS, and gene expression measured on Affymetrix human U133 Plus 2.0 arrays. To identify the genes that correlated with Conners’ Parents Ratings of IA and HI ratings of symptoms, an analysis of covariance (ANCOVA) was performed, controlling for age, gender and batch.

**Results:**

There were 1201 gene probesets that correlated with IA scales, 1625 that correlated with HI scales, and 262 that correlated with both IA and HI scale scores (*P*<0.05, |Partial correlation (*r*_*p*_)|>0.4). Immune, catecholamine and other neurotransmitter pathways were associated with IA and HI behaviors. A number of the identified genes (n=27) have previously been reported in ADHD genetic studies. Many more genes correlated with either IA or HI scales alone compared to those that correlated with both IA and HI scales.

**Conclusions:**

These findings support the concept that the pathophysiology of ADHD and/or its subtypes in TS may involve the interaction of multiple genes. These preliminary data also suggest gene expression may be useful for studying IA and HI symptoms that relate to ADHD in TS and perhaps non-TS participants. These results will need to be confirmed in future studies.

## Background

Inattentiveness, impulsivity and hyperactivity are common behaviors seen in children. When pronounced, these behaviors may lead to the diagnosis of attention-deficit hyperactivity disorder (ADHD) [1]. ADHD is among the most common of the childhood onset psychiatric disorders [2,3]. Clinically, children with ADHD may be diagnosed as predominantly inattentive type, predominantly hyperactive/impulsive type or combined type characterized by both inattention (IA) and hyperactivity/impulsivity (HI) behaviors [3,4]. The difference between the subtypes is based mainly on clinical profiles [4].

Tourette syndrome (TS), characterized by motor and vocal tics, is often associated with ADHD symptoms. TS is a heritable, complex genetic disorder where multiple genes, each with a modest effect, are postulated to interact with unknown environmental factors to produce the phenotype [5,6]. Patients with TS often display comorbid symptoms of ADHD. Of subjects with TS who visit a physician, as many as 50 to 80% have comorbid ADHD, a rate that is 10 to 20 times that of the general population [5]. In our previous study, a subgroup of patients with TS over-expressed natural killer cell genes in blood, and most of these patients with TS had co-morbid ADHD [7]. These findings stimulated the current study to further examine the relationship of gene expression in blood of patients with TS that also exhibit ADHD behaviors.

Recent studies suggest that ADHD symptoms might best be considered as continuous quantitative traits rather than diagnostic categories [1]. This has arisen in part because candidate gene and genetic linkage studies of the ADHD subtypes have shown conflicting results [1,4]. Therefore, this study of gene expression considers inattention and hyperactivity/impulsivity as continuous variables without regard to the categorical clinical diagnoses of ADHD subtypes. Examining these behaviors in participants with TS might provide more homogeneous phenotypes since TS is highly heritable, and is readily and objectively identifiable. Cytogenetic, linkage and GWAS analyses have uncovered a number of loci and several genetic mutations that are associated with Tourette syndrome. For example, mutation in SLIT and NTRK-like 1 (SLITRK1) can cause TS, and though there are other examples, each only accounts for a small fraction of cases [8,9] Notably, our previous study discovered a set of specific alternatively spliced genes that differentiate TS from controls, suggesting that there may be a shared molecular pathophysiology common to many subjects with TS [10].

Thus, the current study quantified IA and HI behaviors using the Conners’ Parent Rating Scales-Revised (CPRS-R) in a group of participants with TS. The well-validated Conners’ scale is widely used in research and clinical practice to diagnose ADHD and evaluate treatment effects in the disorder [3]. Gene expression was quantified using Affymetrix U133 Plus 2.0 arrays and correlated with the IA and HI scores from the CPRS in the same subjects. Gene expression was measured in whole blood because of its accessibility and because of known interactions between the immune system and the central nervous system [7,10-12].

## Methods

### Participants

All participants with TS were recruited via the Tourette Syndrome Association, clinical referrals, local advertisements, physician referrals, and through the University of California at Davis. The participants were recruited as part of a functional magnetic resonance imaging study of tic severity and cognitive control conducted by Dr. S. Bunge and colleagues [13]. All of the participants with TS were diagnosed based on DSM-IV-TR criteria. Tic severity was assessed based on direct child and parent interview using the Yale Global Tic Severity Scale (YGTSS). The CPRS-R was used to assess ADHD symptoms using continuous, standardized age and gender adjusted CPRS-t scores. The parent ratings are useful and valid as they have the opportunity to observe their children over extended periods of time and in a variety of situations. The scale contains 27 items and is composed of 4 subscales including: Cognitive Problems/Inattention, Hyperactivity, Oppositional and the ADHD Index [3]. A major advantage of the CPRS-R is that it uses a very large normative database (8,000+ children) to support the validity and reliability of it. Furthermore, the standardized data from the CPRS were derived from the means and standard deviations for children with and without ADHD. No clinical diagnosis of ADHD was made in the study. Protocols were approved by the institutional review board at the University of California at Davis. Verbal assent was obtained from each subject and written informed consent was obtained from the parent or legal guardian of each participant.

### Sample collection and RNA isolation

Blood sample collection and RNA isolation were performed as described previously [10]. Whole blood (15ml) was collected from each subject via antecubital fossa venipuncture into six PAXgene Vacutainer tubes (Qiagen, Valencia, CA, USA). These tubes contain a solution that immediately lyses all of the cells in whole blood and stabilizes the RNA without measurable degradation. Blood samples were stored frozen at -70°C until processed.

Total RNA was isolated using the PAXgene Blood RNA Kit (Qiagen) according to the manufacturer’s protocol. RNA quality was assessed using the Agilent 2100 Bioanalyzer (Agilent Technologies Inc., Foster City, CA, USA) and quantified using fiberoptic spectrophotometry (Nanodrop ND-1000, Nanodrop Inc., Wilmington, DE, USA). RNA yielding both an *A*_260_/*A*_280_ absorbance ratio greater than 2.0 and a 28s/18s rRNA ratio equal to or exceeding 1.8 was utilized.

### Affymetrix human genome U133 plus 2.0 Microarray processing

Human Genome U133 Plus 2.0 microarray processing was performed according to the manufacturer’s protocol. The Ovation RNA Amplification System V2 kit and the Ovation® WB Reagent kit (NuGEN, San Carlos, CA) were used to optimize whole blood amplification starting with 50 ng total RNA, the amplified cDNA was fragmented and labeled using NuGEN’s FL-Ovation™ cDNA Biotin Module V2 (NuGEN, San Carlos, CA). Hybridization, washing and scanning were performed according to the Affymetrix Human U133 Plus 2.0 protocols (Affymetrix, Santa Clara, CA).

### Data analysis

We deposited the raw data at GEO under accession number GSE30470 and can confirm all details are MIAME (Minimum Information About a Microarray Experiment) compliant. Raw data (Affymetrix.CEL files) were imported into Partek Genomics Suite 6.4 (Partek Inc., St. Louis, MO, USA). Probe summarization and probe-set normalization were performed using Robust Multi-Chip Average (RMA), which included background correction, quantile normalization, log_2_-transformation and median polish probe set summarization. Principal Components Analysis (PCA) was employed to detect outliers because outliers can have a profound influence on correlation coefficients.

To identify the genes that correlated with the CPRS-R inattention or hyperactivity/impulsivity scales, an analysis of covariance (ANCOVA) was performed, controlling for the effects of age, gender and batch (random effect). Of the ~54,000 probesets on the Affymetrix U133 plus 2.0 array, about 36,000 were analyzed after filtering out the probesets targeting non-annotated transcripts, opening reading frames and hypothetical genes. No probesets met the high stringency of a false-discovery correction for multiple comparisons. Thus, we initially considered a main effect of probesets meeting criteria *P*<0.05 and |r_p_| >0.4, supplemented with a pathway and network over-representation approach. Ingenuity Pathways Analysis (IPA 8.0, Ingenuity® Systems) was used to identify statistically significant functional categories in the data set using a modified Fisher Exact test, with *P*<0.05 considered significant. To further support the pathway-related ADHD genes, those involved in the significant pathways were subjected to a co-expression analysis by first performing gene-gene correlation in Partek, and then hierarchical clustering based on gene-gene correlation coefficients by Genesis (Gene Expression Similarity Investigation Suit) software. Chromosome over-representation was identified using the NIAID/NIH DAVID Bioinformatics Resources (http://david.abcc.ncifcrf.gov).

## Results

### Subject demographics

The mean age of the 21 participants with TS in this study was 10.5 years (SD 2.2, range 7 to 15). There were 17 males (81.9%) and 4 females (18.1%), including 15 persons identifying themselves as Caucasian (71.5%), 2 Hispanic (9.5%), and 4 as Other ethnic category (19.0%). The mean tic severity was 23.4 (SD 8.4, range from 8 to 41). The mean HI rating score was 66.1 (SD15.7, range from 47 to 90), and the average IA rating score was 63.1 (SD 13.8, range from 42 to 82). The HI and IA scores had a normal distribution (Kolmogorov-Simirnov Test, *P*=0.13 for HI, *P*=0.48 for IA). All of the participants were medication naive as per parental reports, except for two participants who had previously taken atomoxetine (Strattera™; Eli Lilly, IN, USA) to treat ADHD symptoms. One of these participants ended medication approximately 1 month before participation in the study. The other stopped taking medication 40 hours before participation.

### Gene expression correlation analysis

**Figure 1:**
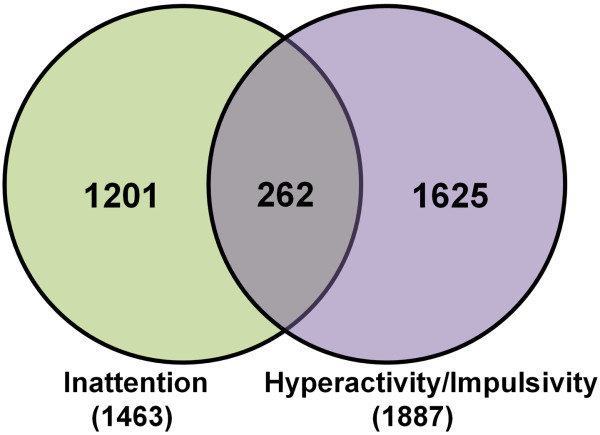
**Venn diagram showing total numbers of probesets that correlated with the CPRS Inattention (IA) and Hyperactivity/Impulsivity (HI) scales or both (*P*<0**.**05, |r**_**p**_**|>0**.**4).** Note that 262 probesets correlated with both the IA and HI scales.

The expression of 1201 probesets (representing 1074 genes) correlated with IA scores (IA-associated genes, *P*<0.05 and |r_p_|>0.4) (Figure 1, Additional file 1: Table S1–1). The expression of 1625 probesets (representing 1364 genes) correlated with HI scores (HI-associated genes, *P*<0.05 and |r_p_|>0.4) (Figure 1, Additional file 1: Table S1–2). The expression of 262 probesets (representing 250 genes) correlated with both IA and HI scores (Common IA-HI genes, *P*<0.05 and |r_p_|>0.4) (Figure 1, Additional file 1: Table S1–3).

#### Common IA-HI associated genes

**Figure 2:**
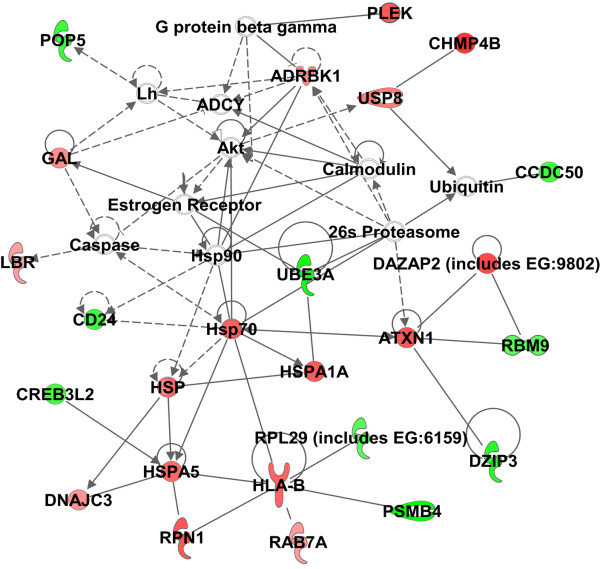
**Network analysis showed cell death**, **behavior and nervous system development and nervous system function as the most over-represented network for the 262 common Inattention/ Hyperactivity/ Impulsivity (IA-HI) probesets (*P*<0**.**05, |r**_**p**_**|>0**.**4).** Red: positively correlated genes, Green: negatively expressed genes.

The over-represented canonical pathways associated with both IA and HI genes included IL-4, B cell receptor, axonal guidance, T cell receptor and glucocorticoid receptor signaling (Table 1, Additional file 2: Table S2–1). An IPA network analysis showed the common IA-HI genes to be associated with cell death, behavior, and nervous system development and function (Figure 2). The co-expression analysis of pathway-related genes revealed distinct patterns of gene expression relating to symptoms. Namely, the genes that positively correlated with both IA and HI scales clustered together, and likewise for the negatively correlated genes (Additional file 3: Figure S1). The common IA-HI genes were over-represented on chromosome 3 (Additional file 2: Table S2–2). Specific genes which have been previously associated with ADHD included solute carrier family 6 (neurotransmitter transporter, noradrenalin), member 2 (*SLC6A2*) and glutamate receptor, ionotropic, N-methyl D-aspartate 2B (*GRIN2B*) (Table 2).

**Table 1:** **Over**-**represented canonical pathways in genes that correlated with CPRS-R for IA**, **HI or both**

**Canonical pathways**	***P***-**value**
**Canonical pathways associated with common IA**-**HI genes**	
IL-4 Signaling	1.10 x 10^-4^
B Cell Receptor Signaling	1.30 x 10^-4^
Axonal Guidance Signaling	9.43 x 10^-4^
T Cell Receptor Signaling	4.95 x 10^-3^
Glucocorticoid Receptor Signaling	8.09 x 10^-3^
**Canonical pathways associated with HI**-**specific genes**	
Integrin Signaling	7.29 x 10^-6^
Toll-like Receptor Signaling	1.61 x 10^-4^
B Cell Receptor Signaling	1.77 x 10^-4^
Role of NFAT in Regulation of the Immune Response	2.71 x 10^-4^
Growth Hormone Signaling	4.71 x 10^-4^
Natural Killer Cell Signaling	8.54 x 10^-4^
**Canonical pathways associated with IA**-**specific genes**	
B Cell Development	2.74 x 10^-5^
Antigen Presentation Pathway	4.93 x 10^-3^
Cardiac β-adrenergic Signaling	7.88 x 10^-3^
B Cell Receptor Signaling	1.02 x 10^-2^
Primary Immunodeficiency Signaling	1.40 x 10^-2^
GM-CSF Signaling	2.15 x 10^-2^

**Table 2:** **Genes that correlated with IA**, **HI scale or both in the current study previously associated with ADHD in published genetic studies**

**Gene symbol**	**Gene title**	***P***-**value** (**HI**)	**r**_**p**_(**HI**)	***P****-***value** (**IA**)	**r**_**p**_ (**IA**)	**Reference**
**Common IA**–**HI genes**						
GRIN2B	glutamate receptor, ionotropic, N-methyl D-aspartate 2B	0.015	−0.54	0.043	−0.47	[14]
SLC6A2	solute carrier family 6 (neurotransmitter transporter, noradrenalin), member 2	0.006	0.58	0.023	0.50	[15]
**HI**-**associated genes**						
BCB1	ATP-binding cassette, sub-family B (MDR/TAP), member 1	0.015	−0.44			[16]
ADAMTS2	ADAM metallopeptidase with thrombospondin type 1 motif, 2	0.048	0.45			[6]
AR	androgen receptor	0.041	0.46			[5]
ARRB2	arrestin, beta 2	0.006	0.62			
COMT	Catechol-O-methyltransferase	0.015	−0.58			[17]
DRD2	dopamine receptor D2	0.007	0.44			[17]
HES1	hairy and enhancer of split 1, (Drosophila)	0.002	−0.60			[17]
LPL	lipoprotein lipase	0.029	−0.52			[6]
MAOA	monoamine oxidase A	0.006	−0.57			[17]
NOS1	nitric oxide synthase 1 (neuronal)	0.004	−0.62			[18]
NR4A2	nuclear receptor subfamily 4, group A, member 2	0.009	0.53			[19]
PPM1F	protein phosphatase 1F (PP2C domain containing)	0.004	0.59			[6]
SLC6A4	solute carrier family 6 (neurotransmitter transporter, serotonin), member 4	0.006	0.65			[17]
SULF2	sulfatase 2	0.013	0.46			[6]
TFEB	transcription factor EB	0.034	0.48			[6]
CCDC136	coiled-coil domain containing 136	0.036	0.409			[20]
ATP11A	ATPase, class VI, type 11A	0.018	0.511			[20]
DUSP1	dual specificity phosphatase 1	0.014	0.551			[21]
SH3BGRL2	SH3 domain binding glutamic acid-rich protein like 2	0.001	0.697			[22]
**IA**-**associated genes**						
FOXP1	forkhead box P1			0.001	−0.62	[6]
FADS2	fatty acid desaturase 2			0.011	−0.52	[23]
DRD1	dopamine receptor D1			0.049	0.44	[24]
MOBP	myelin-associated oligodendrocyte basic protein			0.010	0.48	[6]
PPM1H	protein phosphatase 1H (PP2C domain containing)			0.035	0.47	[20]
PREX2	phosphatidylinositol-3,4,5-trisphosphate-dependent Rac exchange factor 2			0.007	−0.51	[20]

#### HI-associated genes

The over-represented canonical pathways in HI-associated genes included integrin, Toll-like receptor, B cell receptor, role of nuclear factor of activated T-cells (NFAT) in regulation of the immune response, growth hormone and natural killer cell signaling (Table 1, Additional file 2: Table S2–1). The co-expression analysis of pathway-related genes showed separate clustering for the genes that positively and negatively correlated with HI (Additional file 4: Figure S2). The genes were over represented on chromosomes 2, 3, 16, 17, and 19 (Additional file 2: Table S2–2). The genes correlating with HI included catechol-O-methyltransferase (*COMT*), dopamine receptor D2 (*DRD2*), monoamine oxidase A (*MAOA*), and solute carrier family 6 (neurotransmitter transporter, serotonin), member 4 (*SLC6A4*) – all having previously been associated with ADHD (Table 2).

#### IA-associated genes

The over-represented canonical pathways in IA-associated genes included B cell development, antigen presentation pathway, cardiac β-adrenergic signaling, B cell receptor signaling, primary immunodeficiency signaling and GM-CSF signaling (Table 1, Additional file 2: Table S2–1). The co-expression analysis of pathway-related genes showed separate clustering for the genes that positively and negatively correlated with IA (Additional file 5: Figure S3). The genes were over represented on Chromosomes 1, 3, 5, 12 and 13 (Additional file 2: Table S2–2). IA-associated genes which have been previously associated with ADHD included myelin-associated oligodendrocyte basic protein (*MOBP*), dopamine receptor D1 (*DRD1*), forkhead box P1 (*FOXP1*) and fatty acid desaturase 2 (*FADS2*) (Table 2).

## Discussion

This is one of the first studies to relate gene expression in peripheral blood to neuropsychiatric symptoms using whole genome expression arrays. The expression of many genes correlated with the IA, HI scales or both. This finding supports the concept that the pathophysiology of ADHD and/or its subtypes likely involves the interaction of multiple genes. Additionally, the genes that correlated with both IA and HI (common IA-HI genes) may provide a molecular correlate of the combined symptoms in ADHD, as well as facilitate an understanding of the association between IA and HI symptoms. Given the small number of participants, the results are preliminary and will need to be confirmed in subsequent studies. This study did not test whether the genes identified could be used to distinguish individuals with ADHD of the predominantly IA, predominantly HI, or combined types. The current study identified genes that correlated with IA, HI scales or both across all of the participants with TS. These genes might be useful in identifying ADHD phenotypes but future studies with a much larger cohort would be needed to address this question.

### How gene expression in blood might correlate with ADHD symptoms

One of the questions this study raises is how RNA expression in peripheral blood cells might correlate with IA or HI symptoms that are thought to be mediated by central nervous system pathways. First, many of the neurotransmitters and receptors expressed in brain are also expressed in peripheral leukocytes [7,10-12]. Factors that affect neurotransmitters and receptors that mediate symptoms in brain may affect the same neurotransmitters and receptors in leukocytes. Such factors that might affect gene expression in both blood and brain and affect IA and HI symptoms include catecholamines, stress hormones, chemokines and cytokines. In addition, peripheral leukocytes that might be involved in the pathogenesis of TS can signal to neurons via the endothelial cells at the blood brain barrier (BBB). For example, it has been shown that up-regulation of choline acetyltransferase (ChAT) and acetylcholine (ACh) receptor expression in T and B cells [25] can signal via the BBB endothelial cells to neurons in brain [11], a pathway that could modulate ADHD symptoms. Finally, neurons in brain can signal to leukocytes in blood via the endothelial cells at the BBB. For example, neuronal release of catecholamines can signal to BBB endothelial cells which can change adhesion molecule expression on the endothelial cells that then signal to leukocytes. These mechanisms are hypothetical since the current studies cannot gauge what the relationship between blood and brain gene expression might be, particularly given the different genetic influences in blood compared to brain. Though the exact mechanism is unknown, the correlation of gene expression in blood with IA, HI behaviors or both may provide unique insights into pathogenesis of ADHD symptoms.

### Common IA-HI associated genes

Most of the top pathways associated with the common IA-HI genes in participants with TS were immune-related including IL-4 Signaling, B cell receptor signaling, T cell receptor signaling, and glucocorticoid receptor signaling. Glucocorticoid release, which is mediated by the hypothalamic-pituitary-adrenal axis, could affect IA and HI symptoms and gene expression of leukocytes [26]. Network analysis showed the common IA-HI genes were associated with cell death, behavior, as well as nervous system development and function (Figure 2). Imaging studies in ADHD [2,27] have suggested many brain structures associated with cognitive/attention networks display functional abnormalities. These interacting neural regions included the dorsal anterior mid cingulate cortex, dorsolateral prefrontal cortex, ventrolateral prefrontal cortex, parietal cortex, striatum and cerebellum [2]. These brain network changes could be associated at least in part with the molecular network changes noted here (Figure 2).

The neurotransmitter genes *SLC6A2* and *GRIN2B* observed in the common IA-HI gene list have been associated with ADHD. *SLC6A2* is a norepinephrine transporter that has been studied in ADHD due to the fact that drugs that block the norepinephrine transporter are efficacious in treating ADHD [17,28]. SNPs in the *SLC6A2* gene have been associated with ADHD [15]. Glutamatergic signaling pathways also represented candidate susceptibility genes. Thus, three SNPs in the *GRIN2B* gene were associated with ADHD, and quantitative trait analyses showed associations of these markers with both the IA and HI symptom dimensions of ADHD. Disruption of *GRIN1* (2A-D), another glutamate receptor subunit gene, leads to significant alterations in cognitive and/or locomotor behavior including impairments in latent learning, spatial memory tasks and hyperactivity [14].

### HI-associated genes

One of the top canonical pathways over-represented in HI-candidate genes was the role of NFAT in the regulation of the immune response and natural killer cell signaling. This is consistent with a previous report of natural killer cell genes being differentially expressed in TS patients diagnosed with ADHD [7]. Other HI-candidate genes were associated with integrin and growth hormone signaling. Recent Genome Wide Association Studies (GWAS) studies found that basic biological processes, especially integrin signaling, are involved in ADHD pathophysiology [6].

The neurotransmitter-related genes *COMT, DRD2, MAOA* and *SLC6A4* were also included in the HI-candidate gene list and have been previously associated with ADHD [17,29] . *DRD2, COMT* and *MAOA* are catecholaminergic genes. *SLC6A4* is a serotonin transporter that transports the neurotransmitter serotonin from synaptic clefts into presynaptic neurons. *MAOA* is a mitochondrial enzyme which degrades norepinephrine, dopamine and serotonin [17]. *COMT* also catalyzes degradation of catecholamines including dopamine, norepinephrine and epinephrine. The *DRD2* dopamine receptors mediate the effects of dopamine in the indirect basal ganglia pathway. The density of *DRD2* receptors is highest in the basal ganglia, and HI is related to excessive dopamine activity in the basal ganglia [29,30].

### IA-associated genes

Genes expressed in blood that correlated with IA symptoms and have been previously associated with ADHD included *DRD1, MOBP, FOXP* and *FADS2*. *DRD1* is most abundant in the prefrontal cortex (PFC) which is believed to be critical for regulating attention, motivational behavior and emotion. Either too little or too much *DRD1* receptor stimulation impairs PFC function [31]. In addition, genetic studies have suggested an association between *DRD1* with the ADHD IA symptoms in particular [24].

GWAS have suggested that SNPs in the *FOXP1* and *MOBP* genes are associated with ADHD [6]. *FOXP1* is a *FOX* transcription factor family member. FOX transcription factors regulate tissue- and cell type-specific gene transcription during both development and adulthood. Another family member *FOXP2* is involved in developmental speech and language disorders and directly regulates targets related to neural development and synaptic plasticity and developmental disorders like autism and schizophrenia [6].

### Limitations

This study only addressed gene expression correlated with the ADHD symptoms (IA and HI) in participants with TS, and did not consider other co-morbidities like tic severity or obsessive-compulsive symptom severity. It is not known if the genes associated with IA and HI symptoms in the TS subjects could be replicated in general populations of children with ADHD. Given that many genes overlapped between IA and HI symptoms in subjects with TS, some of these might also overlap in subjects with ADHD without TS.

Two participants who had been previously prescribed medication were included in the current study. To determine if these subjects might have biased the results, our Principal Components Analysis (not shown) revealed that there were no outliers in the gene expression data, suggesting these two individuals did not significantly bias the correlations observed. Moreover, our previous studies including these individuals did not show them to be outliers with regard to fMRI findings or alternative splicing [10,13]. Nevertheless, the fact that prior medications might affect blood gene expression should be addressed in future research.

The largest limitation of the study is that, in spite of many genes being correlated with HI and/or IA symptoms, no gene passed multiple comparison correction testing using the Benjamini-Hochberg False Discovery rate (FDR<5%), and none of the genes were confirmed using an independent method such as RT-PCR. Thus, a future confirmatory study likely including RT-PCR and possibly corrections for blood cell types in a much a larger sample size will be needed to validate the genes reported here.

Genetic studies have shown that of the many genes involved in ADHD, a given gene may only contribute a small percent to the symptoms [5,6,17]. This could explain the modest association between a single gene and ADHD symptoms. Thus, pathways identified in this study are likely to be more reproducible in follow up studies rather than individual genes. Importantly, a gene co-expression analysis did validate these pathway-related ADHD genes. Moreover, our gene-gene correlation results demonstrate that the multiple probesets targeting a specific gene on the Affymetrix human U133 arrays were highly correlated each other (Additional file 6 Table S3). The validity of the findings is also supported by the fact that 27 genes that correlated with IA and/or HI scales have been reported in previous genetic studies of ADHD (Table 2).

## Conclusions

These findings support the concept that the pathophysiology of ADHD and/or its subtypes in TS may involve the interaction of multiple genes. Even with limitations, the results suggest a gene expression approach may be useful for defining molecular correlates of IA and HI symptoms in ADHD phenotypes in subjects with TS. A similar approach might be useful in ADHD phenotypes in subjects without TS.

## Abbreviations

ADHD: Attention-deficit hyperactivity disorder; ANCOVA: Analysis of covariance; *r*_*p*_: Partial correlation; TS: Tourette syndrome; DSM-IV: Diagnostic and Statistical Manual of Mental Disorders, Fourth Edition; CPRS-R: Conners’ Parent Rating Scales-Revised; RMA: Robust Multi-Chip Average; *SLC6A2*: Solute carrier family 6 member 2; *GRIN2B*: Lutamate receptor, ionotropic, N-methyl D-aspartate 2B; NFAT: Nuclear factor of activated T-cells; *COMT*: Catechol-O-methyltransferase; *DRD1*: Dopamine receptor D1; *DRD2*: Dopamine receptor D2; *MAOA*: Monoamine oxidase A; *SLC6A4*: Solute carrier family 6, member 4; *MOBP*: Myelin-associated oligodendrocyte basic protein; *FOXP1*: Forkhead box P1; *FADS2*: Fatty acid desaturase 2; ChAT: Choline acetyltransferase; Ach: Acetylcholine; GWAS: Genome wide association studies; GHRs: Transmembrane receptors; JAK2: Janus kinase 2; MAPKs: Mitogen-activated protein kinases; PI3K: Phosphatidylinositol 3-kinases; PKC: Protein kinase C; STATs: Signal transducer and activator of transcriptions; OCD: Obsessive–compulsive disorder.

## Competing interests

The authors declare that they have no competing interests.

## Authors’ contributions

All the authors contributed substantially to the conception and design of the study. YT, JG, BS, BC, BA, CB, NB, PH contributed to the acquisition of data. YT, BS and GJ analyzed and interpreted data. YT,JBS and FRS drafted the manuscript. All authors read and approved the final manuscript.

## Pre-publication history

The pre-publication history for this paper can be accessed here:

http://www.biomedcentral.com/1755-8794/5/49/prepub

## Supplementary Material

Additional file 1 **Table S1.** ADHD symptom related genes. Click here for file

Additional file 2 **Table S2-1.** Canonical pathways with significant over-representation of genes that correlated with CPRS for IA, HI or both (p<0.05, |rp| >0.4). **Table S2**-**2.** Chromosomes significantly over-enriched with genes that correlated with CPRS IA, HI scales or both (p<0.05, |rp| >0.4). Click here for file

Additional file 3 **Figure S1.** Co-expression analysis results of 24 common Inattention/ Hyperactivity/ Impulsivity (IA-HI) pathway-related probesets by using two-way clustering of gene-gene correlation data. Click here for file

Additional file 4 **Figure S2.** Co-expression analysis results of 106 Hyperactivity/ Impulsivity (HI) pathway-related probesets by using two-way clustering of gene-gene correlation data. Click here for file

Additional file 5 **Figure S3.** Co-expression analysis results of 48 Inattention (IA) pathway-related probesets by using two-way clustering of gene-gene correlation data. Click here for file

Additional file 6 **Table S3.** The multiple probesets targted to a specific gene were highly correalted each other. Click here for file
