# Supplementary figures and images for: Correlations of gene expression with ratings of inattention and hyperactivity/impulsivity in tourette syndrome: a pilot study
Source: BMC Med Genomics. 2012 Oct 30;5:49. doi: 10.1186/1755-8794-5-49 (PMC3497583; doi:10.1186/1755-8794-5-49)

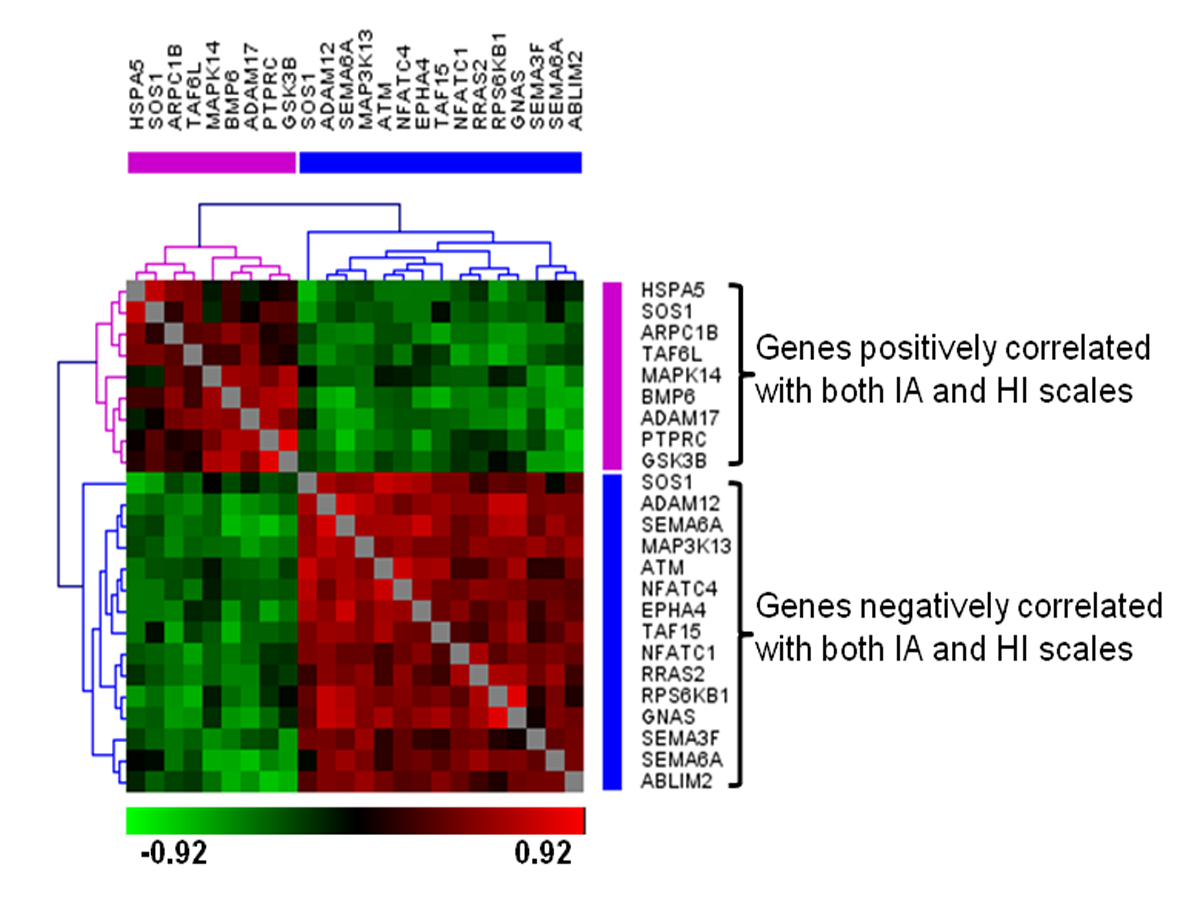

Supplement: Additional file 3 — Figure S1. Co-expression analysis results of 24 common Inattention/ Hyperactivity/ Impulsivity (IA-HI) pathway-related probesets by using two-way clustering of gene-gene correlation data. [file 1755-8794-5-49-S3.tiff]

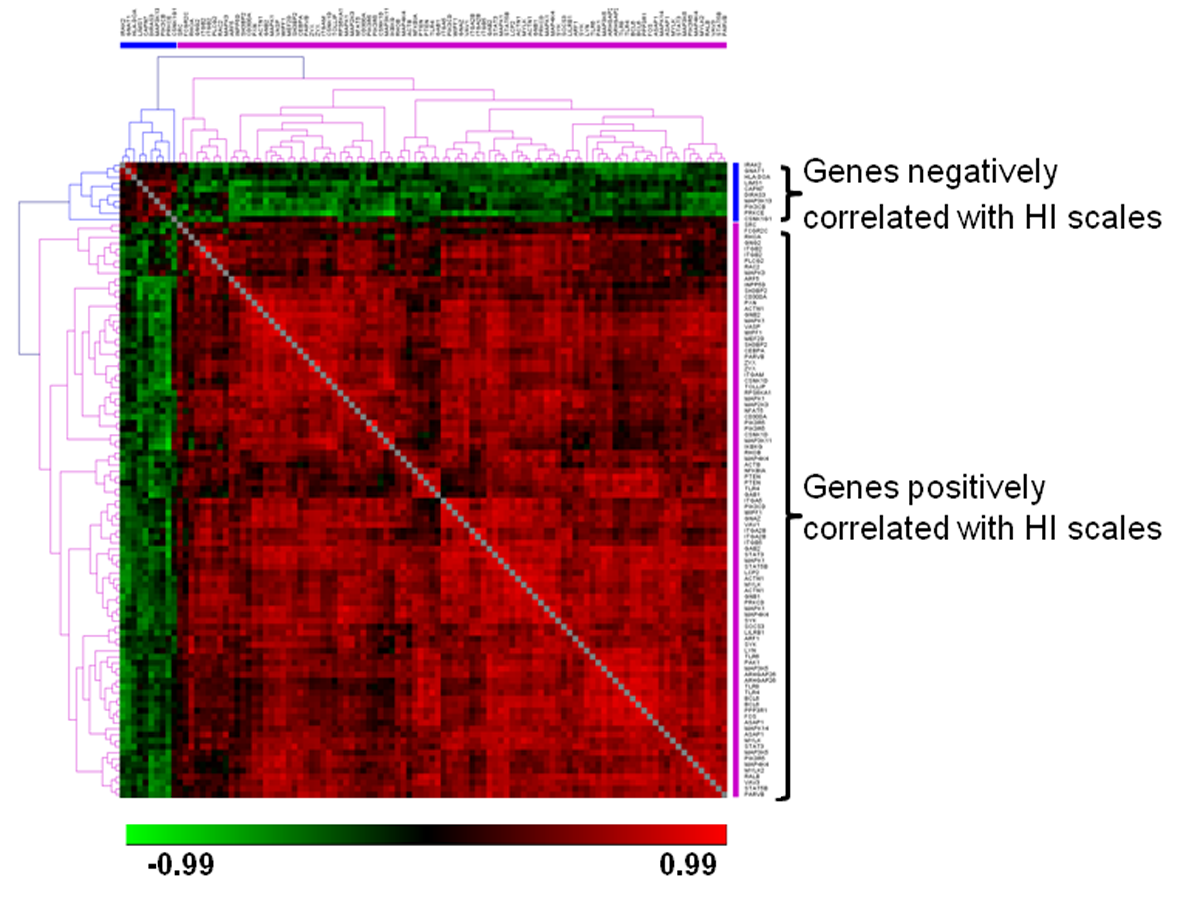

Supplement: Additional file 4 — Figure S2. Co-expression analysis results of 106 Hyperactivity/ Impulsivity (HI) pathway-related probesets by using two-way clustering of gene-gene correlation data. [file 1755-8794-5-49-S4.tiff]

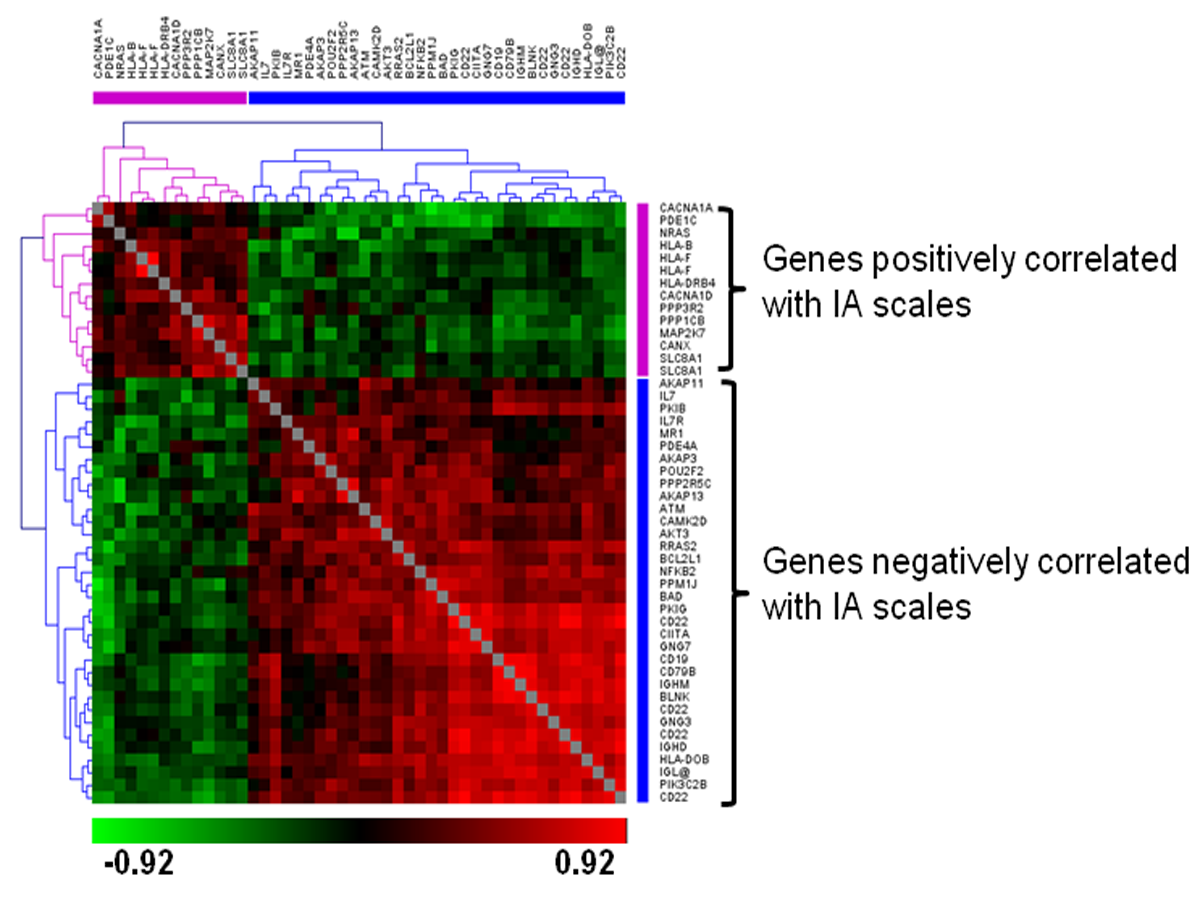

Supplement: Additional file 5 — Figure S3. Co-expression analysis results of 48 Inattention (IA) pathway-related probesets by using two-way clustering of gene-gene correlation data. [file 1755-8794-5-49-S5.tiff]
